# Pesticides and Asthma: Challenges for Epidemiology

**DOI:** 10.3389/fpubh.2014.00006

**Published:** 2014-01-24

**Authors:** André F. S. Amaral

**Affiliations:** ^1^Respiratory Epidemiology, Occupational Medicine and Public Health, National Heart and Lung Institute, Imperial College London, London, UK; ^2^MRC-PHE Centre for Environment and Health, London, UK

**Keywords:** pesticide, insecticide, non-occupational exposure, respiratory health, asthma, air pollution

## Do Pesticides Cause Asthma?

Pesticides are chemical or biological agents used to kill or incapacitate unwanted organisms both indoors and outdoors. In and around the home, these agents are commonly and increasingly used to control rats, ants, cockroaches, flies, moths, mites, weeds, and molds. Pesticides are usually grouped into families not only depending on the organism they target (e.g., fungicides for fungi, insecticides for insects) but also according to their chemical characteristics (e.g., organophosphates, carbamates, pyrethroids).

Asthma is a common chronic inflammatory disease of the airways characterized by variable and recurring respiratory symptoms (wheezing, breathlessness, chest tightness, and dry cough), airflow obstruction, and increased bronchial responsiveness (1, 2). However, it is increasingly recognized that this is not a homogeneous disease since multiple subtypes (e.g., early-onset allergic, late-onset eosinophilic, and exercise-induced asthma) have been described (3). Some of the factors that have been linked to asthma include allergies, tobacco smoking, air pollution, viral and bacterial infections, changes in sex hormone levels, obesity, and occupational exposures (2–5). Variants in several genes (e.g., *CHI3L1, IL6R, DENND1B, IL1RL1–IL18R1, PDE4D, RAD50–IL13, HLA-DQ, IL33, SMAD3, ORMDL3–GSDMB, IL2RB, RORA, TSLP*, and *PYHIN1*) have also been associated with increased susceptibility to this disease (2). Asthma affects more than 300 million people worldwide (6), and in the last decades, its prevalence has been increasing especially in low and middle income countries (7–9). Over the same period, the use of multiple chemicals such as pesticides for crop protection and home disinfestation has also increased (10–12).

Associations between pesticide exposure and asthma in children, adults, and occupational groups have been reported, but these have not yet been shown to be causal and the biological explanation tying the two remains unclear – it could lie in the mechanisms such as irritation (13), inflammation (14), immunosuppression (15, 16), endocrine disruption (17), or a combination of these. So far, it is also unknown how pesticides interact with genes that increase susceptibility to asthma.

## Pesticide Use and Asthma among Farmers

Most of the evidence suggesting a relationship between high levels of exposure to pesticides and asthma come from the studies of farmers. The prevalence of asthma has been associated with the use of pesticides in Canadian and French farmers (18, 19), US farmers and their wives (20, 21), and Australian insecticide applicators (22). Pesticide use has also been associated with wheezing in Kenyan and US farmers (23, 24) and US insecticide applicators (25). In Spain, exposure to low levels of insecticides was associated with a decrease in forced expiratory volume in 1 s (FEV1) in intensive agricultural workers (26).

In a recent publication from the Agricultural Health Study, it is also suggested that the use of insecticides may contribute to the exacerbation of asthma among subjects with allergies (27). Although the translation of these findings to the general population is not straightforward, they do raise concern about the possibility of developing or exacerbating asthma due to prolonged exposures to low levels of pesticides.

## What Do We Know about Pesticide Use and Asthma in the General Population?

Few have studied the role of pesticide use on asthma in the general population. In a small survey of an urban area in the US, 41 out of 181 (22.7%) subjects reported chemical intolerance to at least three of five common chemicals (paint, pesticides, new carpet, car exhaust, perfume). These subjects were more likely to report asthma and respiratory symptoms (wheezing, shortness of breath with wheezing, and chest tightness) than those who tolerated the five chemicals (28). A recent publication from a much larger cross-sectional survey – the US National Health and Nutrition Examination Survey – regarding the association between residential use of pesticides and respiratory symptoms in children showed inconclusive results, i.e., overall pesticide use in the home was not associated with wheezing, but application of pesticides, including insecticides, in the kitchen or dining room was. This exposure was also associated with dry cough (29). Between 2000 and 2008, the incidence rate of non-work-related acute injuries or illnesses caused by pesticides in the US increased from 1.1 to 4.4 per million, with 2,772 out of 3,382 (84%) injuries occurring at home (30). The most common types of exposure were (i) to indoor air contaminated with pesticides and (ii) direct spray exposure to the pesticide during application. Among the subjects exposed to common over-the-counter insecticide sprays and powders, respiratory symptoms (i.e., cough, dyspnea, wheezing, lower respiratory pain, and irritation) were the most frequently reported health effects (30).

## Evidence from a Randomized Study

In a small randomized cross-over study, 25 asthmatic subjects were exposed to low levels of insecticide aerosols and its association with asthma exacerbation was assessed. Compared to the negative control (water), the maximum fall in FEV1 was greater after exposure to standard commercial insecticides (29% of subjects experienced a fall of FEV1 >15%), bronchial responsiveness was more severe, and changes in symptoms affecting the chest, nose, and eyes were also more severe (31). These effects were similar in individuals with mild and severe asthma, suggesting that the risks associated with exposure to insecticides are not limited to subjects in the latter group.

## What are the Limitations of Current Evidence?

The present body of evidence is clearly insufficient to draw conclusions about the effect pesticide use has on asthma. It is uncertain whether pesticides cause asthma or act as triggers for asthma exacerbation or both.

The main limitations of the current evidence are the outcome definition and the exposure assessment. In several studies, the outcome – asthma – was defined by the self-report of respiratory symptoms or doctor diagnosis of disease, lacking information on objective measures of lung function and bronchial responsiveness.

The assessment of exposure is, in most situations, based on self-reports. No biomarker has been used, and study participants have been exposed to a complex mixture of active ingredients and synergists making difficult to point out a specific pesticide or active ingredient, their concentrations, and the time of exposure.

The small size of some of the previous studies and their non-prospective design are other limitations that make difficult to establish a causal relationship between pesticide exposure and asthma.

## Future Directions to Assess the Effect of Pesticides on Asthma

In future studies, asthma definition should be based on the assessment of wheeze, doctor diagnosis, atopy, lung function, and bronchial responsiveness measurements. Detailed phenotyping, including molecular and genetic phenotyping, should be a must-do in future studies. Not only this will strengthen the evidence in favor or against the association between pesticides and asthma but also it will provide insight on the role of pesticides in the various subtypes of asthma.

The continued search by the fast-paced pesticide industry for new active ingredients and new formulations of old active ingredients in combination with more potent synergists is an issue for epidemiologic studies because of the longer time usually needed to set up, perform, and analyze data from such studies. More detailed questionnaires and more focus on individual chemical groups (e.g., pyrethroids), rather than on pesticides grouped by the organisms they target (e.g., insecticides), should be a priority to better assess and understand the potential associations between pesticides and asthma.

Few biomarkers of exposure to pesticides (e.g., serum acetylcholinesterase for exposure to organophosphorus) have been identified, but these in most cases do not provide information on past exposures as they are rapidly metabolized and excreted within hours or days. Because there is yet to be found a good long-term biomarker or set of biomarkers for the different and most commonly used pesticides that would help in understanding the mechanisms behind the potential asthmogenic effects of these agents, the identification and validation of such biomarkers is also a priority.

With larger and preferably prospective studies and better asthma definition and exposure assessment, it will be easier to evaluate the association between individual pesticide exposure and asthma, and additionally it will be possible to identify gene–pesticide interactions. The identification and parameterization of these interactions will mean a big step forward in the understanding of the biological pathways that link pesticide exposure to asthma development and asthma exacerbation.
